# Performance Evaluation of Pavement Geomaterials Stabilized with Pond Ash and Brick Kiln Dust Using Advanced Cyclic Triaxial Testing

**DOI:** 10.3390/ma13030553

**Published:** 2020-01-23

**Authors:** Gaurav Gupta, Hemant Sood, Pardeep Gupta

**Affiliations:** 1 Department of Civil Engineering, National Institute of Technical Teachers’ Training and Research, Sector 26, Chandigarh 160019, India; sood_hemant@yahoo.co.in; 2 Department of Civil Engineering, Panjab Engineering College (Deemed to be university), Sector 12, Chandigarh 160012, India; p_gupta_2000@yahoo.com

**Keywords:** material characterization, resilient modulus, measurement of deformation, geomaterial stabilization, advanced cyclic triaxial testing

## Abstract

An increase in the generation of industrial waste materials such as pond ash and brick kiln dust represents a serious threat to the earth’s environment. The waste materials have novel characteristics and therefore their physical, chemical, mineralogical, morphological, mechanical, and toxicity characteristics were determined so that these materials can be incorporated as construction materials based on technical and environmental considerations. The purpose of this study is to utilize the wastes in the stabilization of clay geomaterial to outperform existing problems of inadequate strength and stiffness of the subgrade layer in flexible pavements. Mixtures of geomaterials, containing pond ash, brick kiln dust, and their combination were prepared and important engineering characteristics such as the plasticity, compaction, and strength of the mixtures were examined. The measurement of response to dynamic loading is a pre-condition for the accurate characterization of geomaterials used in pavement construction. Using advanced cyclic triaxial testing, this study evaluates the performance of pond ash and brick kiln dust in increasing the stiffness of the geomaterial under cyclic loading. To stimulate the worst field conditions, the stiffness and strength tests were performed under standard and four-day water-soaked conditions. The implementation of several stress-dependent models for the prediction of stiffness was examined. Pond ash and brick kiln dust were found to be effective in increasing the stiffness and strength of the geomaterial. The wastes were the most effectual when added in combination to the geomaterial. The characterization of the wastes was informative in understanding the governing mechanism prevalent in the waste stabilized mixtures. The toxicity characterization study revealed the non-toxic and non-hazardous nature of the waste materials, permitting their use in the construction of pavements. This study recommends the use of wastes in the subgrade of flexible pavements. Further research is needed for performance evaluation of the wastes on silt and sand geomaterials for their wider application.

## 1. Introduction

More than 70% of the electricity and 60% of the industrial energy in India comes from coal-fired thermal power plants [1,2]. Until replaceable energy production methodologies are adopted, coal will persist to be the chief fossil fuel for India. India accounts for 9.6% of the total coal reserves (1054.8 BT) in the world out of which 95.2% are anthracite and bituminous coal and 4.9% are sub-bituminous and lignite coal [3]. The combustion of coal leaves behind a large volume of fly ash and bottom ash. Water is added to the wastes to form a slurry that is pumped to nearby open areas to form ash ponds [4]. By the middle of 2019, national thermal power plants in India collectively reported a stock deposit of more than 540 million tonnes of unutilized pond ash that has engaged more than 113 million square meters of land, making it redundant for any use [5,6]. Solid fired clay bricks are among the most widely used building materials in the world. With 11.3% share in the worldwide production of bricks, India produces the second-highest number of bricks in the world [7]. India houses 1.4 million brick kilns that are already producing 250 billion bricks annually with a 2% to 5% growth in production every year [8,9]. The conventional practice of firing clay bricks involves burning of large quantum of wood and coal for fuel, combustion of which results in the production of brick kiln dust, a mixture of burnt soil, coal ash, wood ash and brickbats [10]. The prevalent practice of dumping of brick kiln dust in open areas has resulted in its identification as the second most considerable air pollutant in India, following fly ash [10]. The increase in generation of pond ash and brick kiln dust has led to a scarcity of land for their disposal and it is enormously dangerous to society hygiene and environment. The leachate emanating from the wastes contain heavy metals and toxic elements that may contaminate land, groundwater and surface water bodies [11,12,13,14]. On the other hand, the expansion of the road network is of fundamental significance in the socio-economic advancement of a nation. The subgrade is the lowest layer in a roadway pavement and is usually built out of locally available geomaterial deposits that are soft/weak and do not possess adequate stiffness to support repeated traffic loading, plying on the pavement surface. Substitution of weak geomaterials with enhanced stiffness/strength borrow geomaterials/fill may not be cost-effective and logistically feasible because of the related additional expenditure of excavation and the transportation of the materials. Soil stabilization is a well-known technique for enhancing the engineering characteristics of the clay soil such as improved durability, stiffness and strength with minimized shrinkage, swelling and plasticity [15,16]. Bulk utilization of pond ash and brick kiln dust in stabilization of weak geomaterials can circumvent the problems related to disposal of solid waste and poor stiffness of the geomaterial, provided the novel characteristics of the waste establish technically sound and environmentally safe applicability of the wastes.

Seed et al. were the first to conceptualize resilient modulus (M_R_) as material stiffness for illustrating the elastic response of geomaterials to repeated traffic loading [17]. Major pavement design guides identify M_R_ as a chief structural input for the design of pavements using mechanistic-empirical approach and mandate determination of M_R_ through laboratory advanced cyclic triaxial testing [18,19,20].

Improved mechanical characteristics of pond ash and brick kiln dust stabilized geomaterials have been reported in the literature under static loading tests. The increase in the California bearing ratio (CBR) of variable plasticity was reported to be the range of 80% to 600% and 66% to 350% with pond ash and brick kiln dust stabilization respectively [21,22,23,24,25]. The M_R_ of geomaterials stabilized with wastes such as fly ash, cement kiln dust, oil shale ash, bottom ash, municipal solid waste ash, and ground granulated blast furnace slag have been reported in literature [26,27,28,29,30,31,32]. The stabilization process led to a noteworthy increase in M_R_ of the geomaterials. The M_R_ was found to be dependent on cyclic deviator stress, confining pressure and the moisture content of the specimen. However, no literature is available on the resilience response of pond ash stabilized or brick kiln dust stabilized geomaterials.

In this paper, important characteristics of the wastes, such as physical, chemical, mineralogical, morphological and mechanical were investigated to understand the governing mechanisms and controlling factors affecting the M_R_ of waste stabilized geomaterials. The waste materials used were pond ash and brick kiln dust. Toxicity characteristic leachate procedure was adopted to obtain leachate from pond ash and brick kiln dust. The leachates were tested for the concentration of heavy metals to seek their potential to contaminate land and water. The plasticity limits, compaction characteristics and strength characteristics of the geomaterial stabilized with separate and combined dosage of the wastes were determined. Advanced cyclic triaxial tests were carried out to investigate the performance of pond ash and brick kiln dust stabilized geomaterials under the action of repeated traffic loading. The effect of cyclic deviator stress and confining pressure was found in the study. The implementation of several stress-dependent models for the prognosis of M_R_ was examined.

The major contributions of the current research are highlighted here under

- An in-depth characterization of the waste materials was performed for understanding the governing mechanism responsible for imparting strength and stiffness to the waste stabilized geomaterial. Safe application of the waste was ensured by testing leachate emanating from the wastes for concentration of toxic elements.
- The performance evaluation of pond ash and brick kiln dust stabilized geomaterial was done using advanced cyclic triaxial testing to stimulate field conditions in the laboratory.

## 2. Test Materials

Pond ash was collected from the ‘Deenbandhu Chhotu Ram Thermal Power Station’ at Yamunanagar in Haryana state of India. Brick kiln dust was procured from a brick kiln located at Mohali in Punjab state of India. Clay geomaterial was gathered from Chandigarh in India. The physical characteristics of the raw materials have been reported in Table 1. Unified soil classification system (USCS) was followed for the classification of the raw materials. The parameter *D_x_* in Table 1, denotes that x% of the material particles are finer than the size of the reported value.

## 3. Test Methods

The test methods used for the characterization of pond ash and brick kiln dust, optimization of the dosage of the wastes for stabilization of the geomaterial, and advanced cyclic triaxial testing is discussed in the following sections. For each mix ratio parameter, three parallel trials were carried out for each of the test methods and the average of the results have been reported in the figure where the error bars span one standard deviation from the average value [33,34]. The standard deviation is calculated from the average of the three-experimental readings, and therefore the error depicted through error bars depends upon the results of three parallel trials of each mix ratio.

### 3.1. Characterization of Waste Materials

Physical characteristics of the wastes such as particle size distribution and specific gravity were determined in accordance with standard procedures such as dry and wet sieve analysis (including hydrometer analysis) for the determination of the particle size distribution and pycnometer method for the determination of the specific gravity of the wastes. Grain size distribution was obtained by carrying out sieve analysis, where the sample was made to pass sieves having an aperture size in the range of 4.75 mm to 75 μm. The samples passing below the 75 μm sieve was subjected to hydrometer analysis. The size of particles finer than 60%, 30%, and 10% were used to determine the gradation of samples. The compaction and strength characteristics of pond ash and brick kiln dust were determined through Proctor test (modified) and CBR test in accordance with IS 2720 Part 8 and Part 16 [35,36]. The chemical compositions of pond ash and brick kiln dust were investigated using a wavelength dispersive X-Ray fluorescence (WDXRF) spectrophotometer (Bruker, model: S4 Pioneer). Loss on ignition (LOI) test was carried out in accordance with IS 1917 [37]. The mineralogical characteristics of pond ash and brick kiln dust were investigated using an X-ray diffraction spectrometer (Make: PANAnalytical, Model: X’Pert Powder). The crystalline phases were identified across a range of 10°to 70° scattering angle, using ‘HighscorePlus’ software. The morphological characteristics of pond ash and brick kiln dust were investigated using a scanning electron microscope (SEM) system (Make: JEOL, Model: JSM-6100). Due to the non-conductive nature of the samples, a sputter coater (Make: JEOL, Model: JFM 1100) was used to coat a thin layer of gold-palladium alloy on the samples to make them good conductors. For obtaining the toxicity characteristics of pond ash and brick kiln dust, the leachate of the samples was obtained through toxicity characteristic leachate procedure method as per the methodology mandated by United States Environmental Protection Agency (USEPA) [38]. The leachates were investigated for the concentration of heavy metals such as lead (Pb), mercury (Hg), chromium (Cr), arsenic (As), zinc (Zn), nickel (Ni) and copper (Cu) and cobalt (Co) by the means of MP-atomic emission spectrophotometry.

### 3.2. Engineering Characteristics of Waste Stabilized Geomaterial

Laboratory investigations were conducted on the geomaterial stabilized with separate and combined dosage of wastes. The percentages used for pond ash and brick kiln dust were 10%, 20%, 30%, and 40% of the total mass of the mixture. The mix proportions for the geomaterial stabilized with combinations of pond ash and brick kiln dust have been summarized in Table 2, where PA and BKD correspond to pond ash and brick kiln dust respectively. Literature reports that the optimum content of pond ash and brick kiln dust lies between 30%–40% [21,22,23,24,25]. Plasticity characteristics such as liquid limit, plastic limit and plasticity index were investigated as per IS 2720 part 5 [39]. Compaction tests (modified Proctor) were performed as per the standard codal provisions (in five layers) to develop a relationship between density and moisture. Using the plot, maximum dry density (MDD) and optimum moisture content (OMC) were determined. CBR of the samples was investigated using a static load compression testing machine. The rate of strain was maintained at 1.25 mm/min during the CBR testing. The samples were compacted in a static loading press, to attain 97% of MDD at (OMC), obtained through heavy Proctor compaction test, satisfying the criterion for the minimum density recommended by IRC 37 [20]. The compaction was carried out using static compaction as the flexible pavement design guidelines IRC 37 [20] recommend static compaction over dynamic compaction to achieve a more uniformly compacted sample for CBR test. In the static compaction process, dry loose soil was mixed with moisture corresponding to OMC and the mass of this soil, required to achieve 97% of the MDD was filled in the CBR mould, followed by tamping to remove the air voids. Thereafter, a filter paper was placed on the soil, upon which a spacer disc of 50 mm thickness was placed. The assembly was compressed in a compression testing machine such that the top of the spacer disc is flush with the top of the collar of the CBR mould. The load is then held for 30 seconds and then released. For soaking, the samples were immersed in water for a period of four days (96 hours) prior to testing in accordance with IRC 37 [20]. The contents corresponding to maximum CBR value were adopted as the optimum dosage.

### 3.3. Advanced Cyclic Triaxial Testing for Determination of Resilient Modulus

Advanced cyclic triaxial tests were performed using the dynamic triaxial testing machine (Geotechnical Digital System, Model: ELDYN, UK). Prior to the start of the testing, the sample was subjected to 500–1000 cycles of loading for its pre-conditioning. Pre-defined in 15 stress stages, M_R_ testing is carried out at variable cyclic deviator stress and confining pressure. For each stage, 100 repetitions are applied to the test specimen in accordance with the pre-defined stresses illustrated in AASHTO T-307, reported in Table 3 [40].

The duration of loading in laboratory testing should simulate the stress pulse generated by the traffic loading on pavements occurring in real-time [41]. AASHTO T-307 states that the cyclic loading shall comprise repeated haversine-shaped load pulse, wherein the load is applied for a period of 0.1 s preceded by a no-loading period of 0.9 s. The applied cyclic deviator stress is measured by means of an internally submersible load cell. For each loading stage, the recoverable deformation is measured by two externally affixed linearly variable differential transducers (LVDT). The measured recoverable deformation during the last five cycles is averaged and used to determine the M_R_. This results in variable M_R_ values determined from each of the 15 loading cycles due to the non-linear stress-strain behaviour of the geomaterial. The cell pressure was attained through the use of compressed air. An external pressure transducer was used for accurate measurement of cell pressure. The sample preparation for the test comprises casting of cylindrical specimens with a height of 142 mm and a height to diameter ratio of 2. In order to stimulate the worst field conditions, four-day water-soaked samples were tested in addition to the unsoaked samples.

## 4. Results and Discussion

### 4.1. Characteristics of the Wastes

#### 4.1.1. Physical

**Figure 1:**
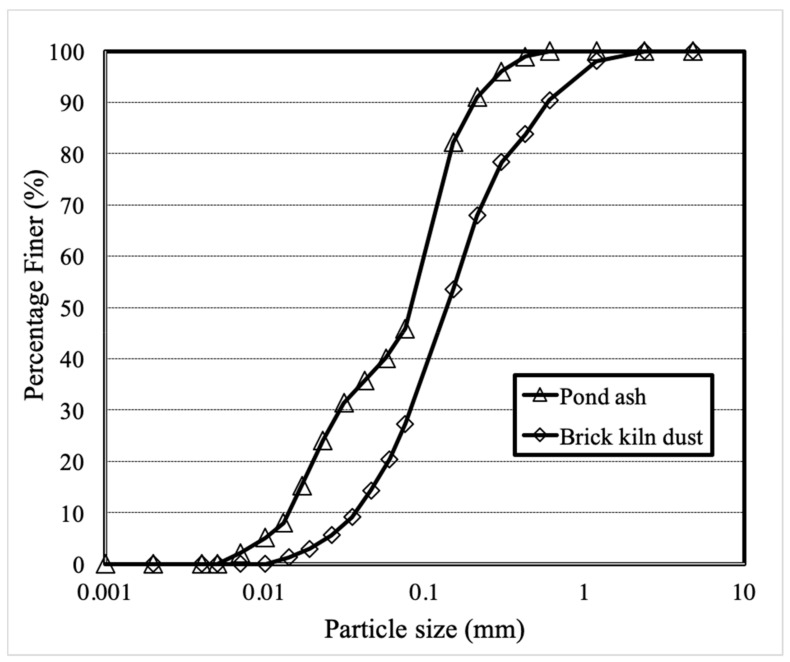
Particle size distribution curve for pond ash and brick kiln dust.

The specific gravity of pond ash and brick kiln dust was found to be 2.0 and 2.20 respectively and lower in comparison with the range of specific gravity of standard geomaterials of 2.5 to 2.7 characteristics [42]. This behaviour could be attributed to the presence of cenospheres in the wastes [42]. Higher specific gravity of brick kiln dust than pond ash could be due to coarser particle size and higher iron content in brick kiln dust [43,44]. The grain size distribution curves of pond ash and brick kiln dust samples have been reported in the Figure 1. Pond ash and brick kiln dust were found to be poorly graded materials and hence are suitable for use as a fill material and improve the gradation of weak geomaterials through stabilization of geomaterials. The investigation shows that the major portion of pond ash and brick kiln dust are coarser particles i.e., sand size particles with some silt size particles. Based on the unified soil classification system (USCS), pond ash and brick kiln dust were categorized as sand with an appreciable amount of silt (SM). It was seen that the current results fall in the range of Indian coal ashes [42,45].

#### 4.1.2. Chemical

The chemical composition of pond ash and brick kiln dust is reported in Table 4. Both pond ash and brick kiln dust are primarily composed of quartz, alumina, and iron oxide, along with a minute amount of oxides of calcium, titanium, magnesium, phosphorous, potassium, sulphur, sodium and manganese. These findings were in coherence with the literature [42,43,44,45,46,47,48]. Brick kiln dust comprises higher iron content and lower quartz content than pond ash. High content of quartz and alumina content impart good potential bonding ability of the wastes [49]. By reason of the low proportion of calcium oxide, the cementing properties of the waste are bound to be negligible. Table 4 shows that the sum of proportion of quartz, alumina, and iron oxides present in pond ash and brick kiln dust was above 70%, and therefore the waste materials may possess pozzolanic properties in accordance with ASTM C618 [50]. However, owing to their coarser particle sizes, the wastes do not full fill the criteria of physical characteristics for utilization as a pozzolanic material. As the waste materials lack cementing or pozzolanic properties, curing of the samples containing these wastes would not result in an increase of stiffness and strength characteristics. Therefore, in the present study, all specimens containing the wastes were subjected to laboratory testing immediately after sample preparation. The LOI values of pond ash and brick kiln dust were determined as 2.1% and 1.1% respectively. The values being lower than 5%, ensure the elimination of any risk of spontaneous or self-ignition heating of the wastes.

#### 4.1.3. Minerological

**Figure 2:**
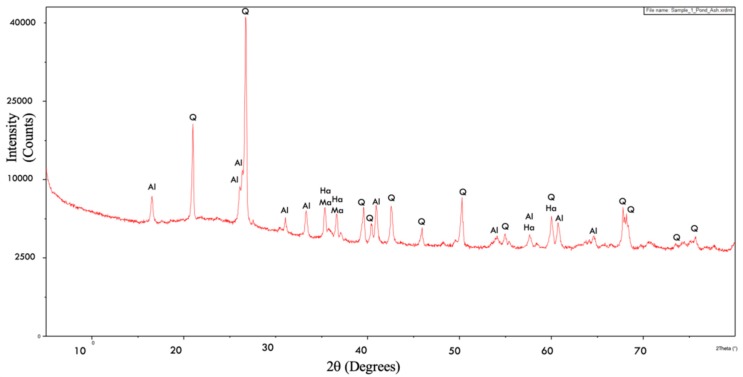
XRD diffractogram for Pond ash.

The diffractogram of pond ash is shown in Figure 2. The type of minerals and their proportion present in the raw materials is pivotal in controlling the mineralogy of the products generated after their combustion. It was observed that quartz (SiO_2_)-Q and mullite (3Al_2_O_3_.SiO_2_)-Al were the predominant minerals present in pond ash accompanied by hematite (Fe_2_O_3_)-Ha and magnetite (Fe_3_O_4_)-Ma in minor proportions. The peaks of quartz were noticed at 20.9°, 26.7°, 36.6°, 39.6°, 40.3°, 42.5°, 45.9°, 50.3°, 54.9°, 60.0°, 67.8°, 68.2°, 73.5°, and 75.6°positions. The peaks of mullite were identified at 16.3°, 26.1°, 26.3°, 31.1°, 33.3°, 40.9°, 54.0°, 57.6°, 60.7°, and 64.6°positions. Peaks of hematite were found at 35.3°,37.1°,57.6°, and 60.0°, where magnetite was also noticed at 35.3° and 37.1°positions. These findings were in coherence with the XRD diffractograms of pond ash reported in literature [44,45].

**Figure 3:**
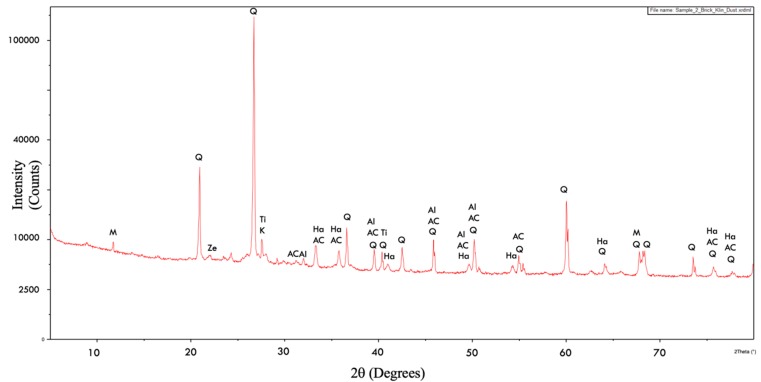
XRD diffractogram for brick kiln dust.

The diffractogram of brick kiln dust is shown in Figure 3. The major minerals present in brick kiln dust were quartz (SiO_2_)-Q and hematite (Fe_2_O_3_)-Ha, calcium aluminium silicide (Al_2_Ca_3_Si_2_)-AC and aluminium oxide (Al_2_O_3_). Small amount of zeolite-Ze, potassium-K, magnesium-M and titanium-Ti oxides were also present. The peaks of quartz were noticed at 20.9°, 26.7°, 36.6°, 39.6°, 40.3°, 42.5°, 45.9°, 50.2°, 54.9°, 60.0°, 64.1°, 67.8°, 68.2°, 73.5°, 75.7°, and 77.8°positions. The peaks of calcium aluminium silicide were noticed at 26.7°, 31.3°, 33.3°, 35.8°, 39.5°, 45.9°, 49.6°, 50.2°, 54.9°, 75.7°, and 77.7°positions where aluminium oxide was also observed at 39.5°, 45.9°, 49.6°, and 50.2°. The peaks of hematite were noticed at 35.7°, 40.9°, 49.6°, 54.3°, 64.1°, 75.7°, and 77.8°. These findings were in coherence with the XRD diffractograms of brick kiln dust reported in literature. [46,47].

Quartz has a relatively elevated melting point of 1400°C and therefore majority portion of the mineral remains in pond ash and brick kiln dust. Being non- reactive at routine temperatures, existence of quartz in a major fraction tend to mitigate the reactivity of the wastes. Therefore, it can be concluded that pond ash and brick kiln dust behave like an inert material and can be safely used as construction materials. It may be noted that as brick making soil contains higher iron content, hematite was a major mineral identified in brick kiln dust whereas it was present in minority in the pond ash sample. The XRD results were found in agreement with the XRF results, where brick kiln dust had higher iron and calcium content and lower quartz and alumina content than in pond ash. Reduced quartz and alumina content and higher calcium content could be due to lower furnace temperature of brick kilns than thermal power plants and the presence of additional minerals in burnt clay soils from brick kilns.

#### 4.1.4. Microstructural

**Figure 4:**
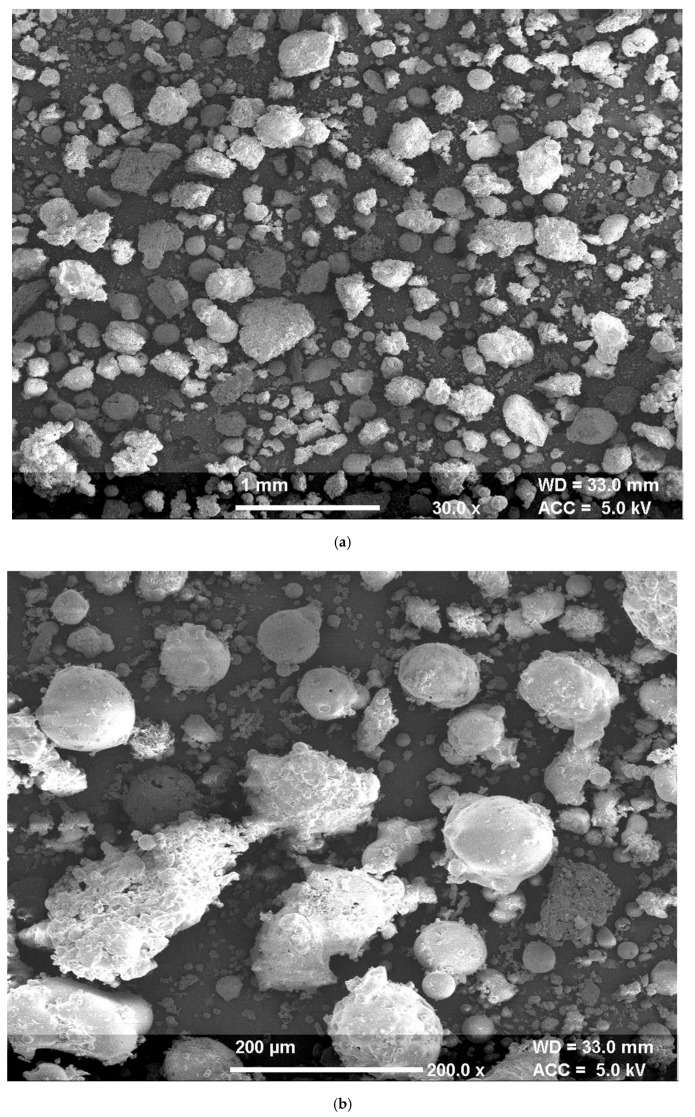
(**a**): SEM micrographs for pond ash at magnification 30×; (**b**): SEM micrographs for pond ash at magnification 200×.

The SEM micrographs for pond ash at 30× and 200× magnifications are presented in Figure 4a,b respectively. SEM micrographs show a round shape and a smooth surface of pond ash particles. Further at higher magnifications, the micrographic observations indicate agglomeration of fine particles and sticking of fine particles to the coarser particles. The micrograph at 200× magnification shows that certain large particles of pond ash comprise a bundle of tinier round particles. These findings were in coherence with the SEM micrographs of pond ash reported in literature [42,43].

**Figure 5:**
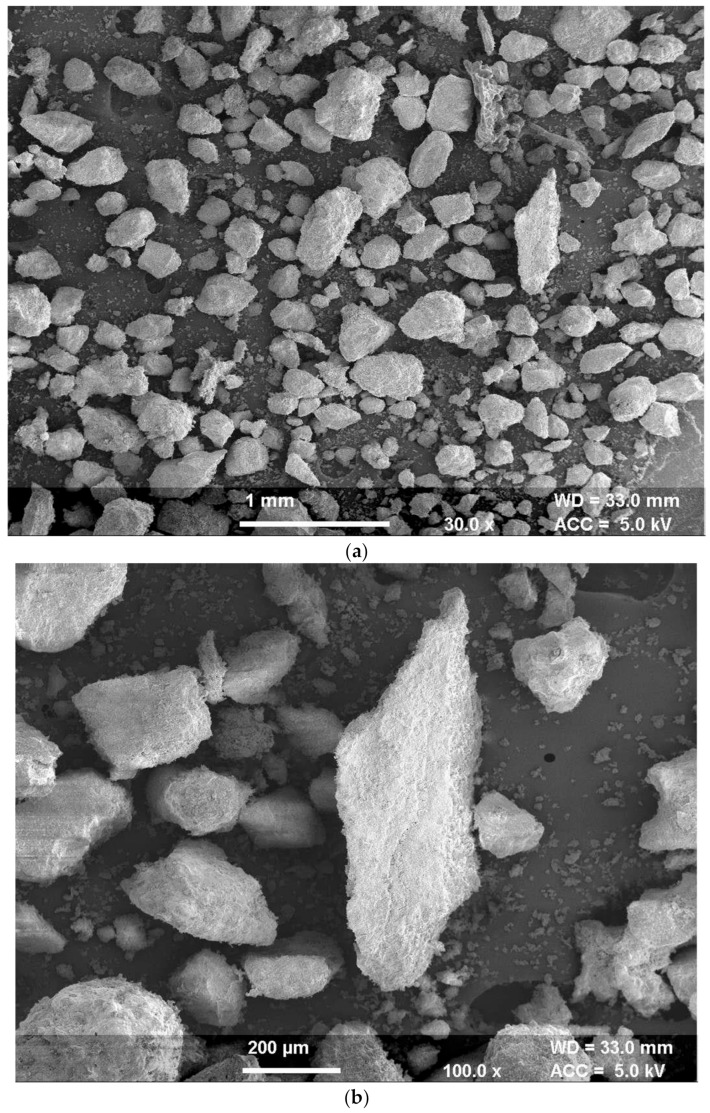
(**a**): SEM micrographs for brick kiln dust at magnification 30×; (**b**): SEM micrographs for brick kiln dust at magnification 100×.

The SEM micrographs for brick kiln dust at 30× and 100× magnifications are presented in Figure 5a,b respectively. SEM micrographs show an irregular structure with a flaky shape and a rough surface of brick kiln dust particles. Further at higher magnifications, the micrographic observations indicate an agglomeration of variable sized granules with scattered empty spaces. These findings were in coherence with the SEM micrographs of brick kiln dust reported in the literature [44,45].

#### 4.1.5. Mechanical

The mechanical characteristics of pond ash and brick kiln dust samples such as compaction, bearing strength and permeability were determined. In comparison with the standard range of MDD and OMC for geomaterials, the MDD values of the waste were found to be lower whereas the OMC values of the waste were found to be higher. Comparing the compaction characteristics of pond ash and brick kiln dust, MDD of pond ash was found to be lower whereas the OMC value of pond ash was found to be higher. Reduction in MDD is attributed to reduced specific gravity of the wastes as pertinent from results reported previously in Table 5. Pond ash and brick kiln dust are bound to resist external compactive effort due to the development of shear resistance in between the particles of the wastes and development of pore water pressure during compaction of the waste. Rise in OMC is attributed to increased fineness and presence of un-burnt carbon in the wastes and also due to have different water absorption capacities of the wastes. As per the specifications (5th Revision) issued by Ministry of Road Highways and Transport, Government of India (MORTH) for construction of roads in India, the criteria for the minimum density of the material forming the subgrade material i.e., 17.5 kN/m^3^ under modified effort, was not achieved with either of the wastes [51]. Therefore, pond ash and brick kiln dust cannot be used in totality in the construction of the subgrade of pavements.

The bearing strength of pond ash and brick kiln dust are reported in Table 5. In comparison with the standard range of CBR for clay geomaterials, the CBR of the wastes were found to be higher. Increase in CBR is attributed to absence of plasticity in the wastes. Comparing the CBR of pond ash and brick kiln dust, the CBR of pond ash was found to be lower due to lower sand content than brick kiln dust and round shape of its particles that have reduced interlock friction. Higher CBR under unsoaked conditions was reported because of additional capillary forces present in partly saturated condition. Immersion in water nullifies the capillary forces, as an effect of which the value of CBR falls.

The values of coefficient of permeability of pond ash and brick kiln dust are reported in Table 5 and were found to be equivalent to those of silts. The values of coefficient of permeability indicate that both the wastes allow free drainage of water and can be effectively used in backfilling of retaining walls and road embankments.

#### 4.1.6. Toxicity

The concentrations of toxic elements determined in the leachate of pond ash and brick kiln dust are reported in Table 6 along with the allowable limits for hazardous waste as stipulated by the USEPA. Zinc and lead were identified in the leachate of pond ash whereas zinc, lead and arsenic were identified in the leachate of brick kiln dust. All other toxic elements such as chromium, copper, cadmium, mercury, and nickel were found below the atomic absorption spectrophotometer’s detection limit of 0.1 mg/L. In totality, the concentrations of toxic elements were determined to be well within the allowable limits, and hence the use of pond ash and brick kiln dust in construction applications is non-hazardous and does not pose a threat to the environment.

### 4.2. Engineering Characteristics of Waste Stabilized Geomaterial

#### 4.2.1. Plasticity

**Figure 6:**
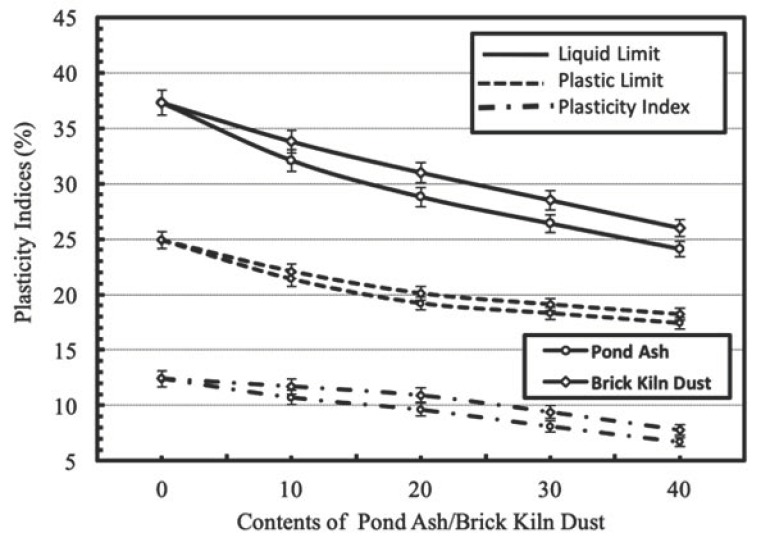
Variation in plasticity indices with varying contents of pond ash or brick kiln dust.

**Figure 7:**
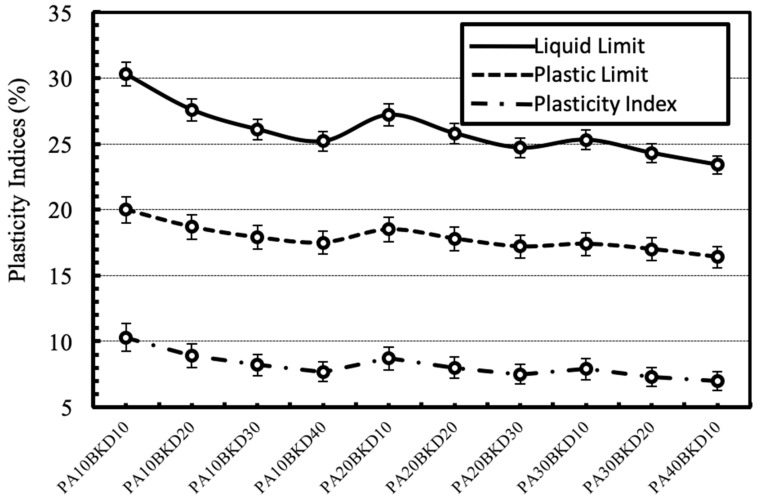
Variation in plasticity indices with varying combinations of pond ash and brick kiln dust.

Three plasticity characteristics such as liquid limit, plastic limit and plasticity index, which are the indices for quantifying the activity of clay minerals, were used to determine the effect of stabilization on the plasticity of the geomaterial. The effect of the separate and combined dosages of the wastes on the plasticity indices of the geomaterial is shown in Figure 6 and Figure 7 respectively. In general, plasticity decreases with the increase in contents of pond ash and brick kiln dust. This may happen due to the addition of non-plastic particles of pond ash and brick kiln dust that leads to a reduction of the plasticity of the stabilized mixtures. It is expected that reduction in plasticity of the stabilized mixes would result in an improvement of their strength characteristics.

#### 4.2.2. Compaction

**Figure 8:**
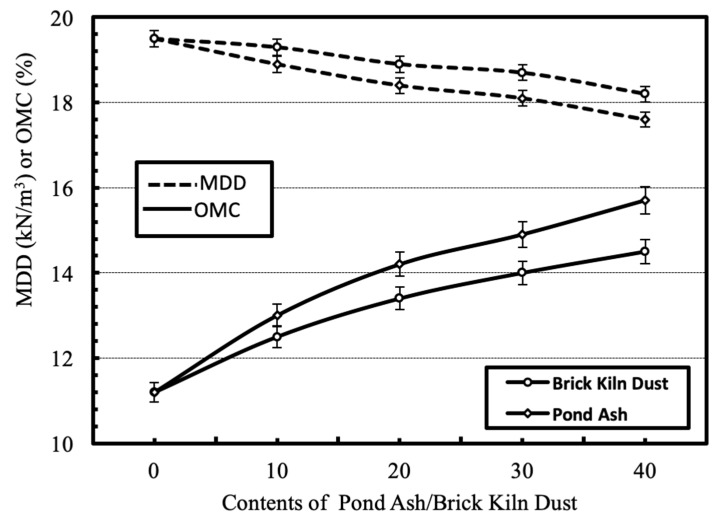
Variation in compaction characteristics with varying contents of pond ash or brick kiln dust.

**Figure 9:**
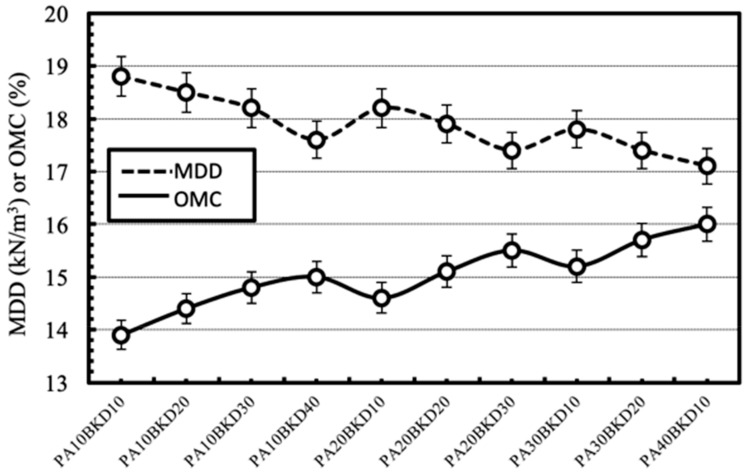
Variation in compaction characteristics with varying combinations of pond ash and brick kiln dust.

The effect of separate and combined dosages of the wastes on compaction characteristics i.e., MDD and OMC, of the geomaterial is shown in Figure 8 and Figure 9 respectively. In general, MDD of the geomaterial decreases and OMC of the geomaterial increases with increase in contents of pond ash and brick kiln dust. The decrease in MDD may be due to comparatively lesser specific gravity of the waste than the geomaterial. The increase in OMC may be due to the comparatively higher fineness of the wastes than the geomaterial and presence of unburnt carbon in the wastes. The effects were higher for pond ash than brick kiln dust due to lower specific gravity and higher fineness of pond ash particles. The combination of pond ash and brick kiln dust further decreases the MDD and increases the OMC of clay geomaterial. Similar trends have been reported in literature [22,23,24,25].

#### 4.2.3. Strength

**Figure 10:**
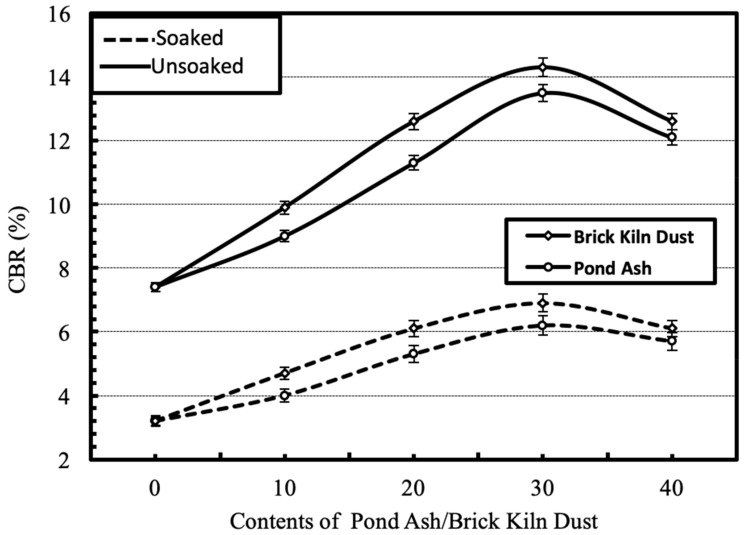
Variation in CBR of the geomaterial with varying contents of pond ash or brick kiln dust under soaked and unsoaked conditions.

**Figure 11:**
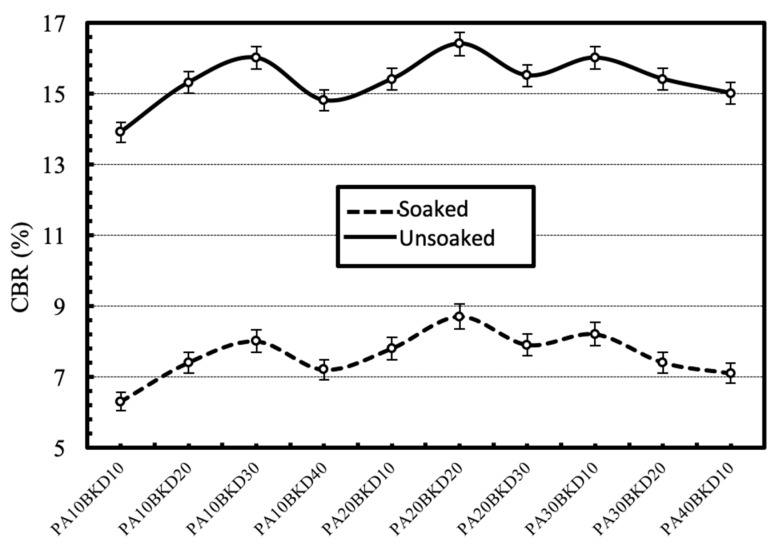
Variation in CBR of the geomaterial with varying combinations of pond ash and brick kiln dust under soaked and unsoaked conditions.

The effect of separate and combined dosages of the waste on the strength characteristics, i.e., unsoaked and soaked CBR of the geomaterial is shown in Figure 10 and Figure 11 respectively. The CBR of the geomaterial increases upto the addition of 30% pond ash or 30% brick kiln dust, beyond which it decreases. The increase in CBR was higher for brick kiln dust than pond ash. The combined action of pond ash and brick kiln dust stabilization further improved the CBR but only upto a combination of 20% brick kiln dust and 20% pond ash. The increase in strength is attributed to improved frictional strength, minimized plasticity and enhanced gradation of geomaterial-waste mixture comprising of sand, silt and clay particles. The reduction in CBR due to soaking was reported to fall in the range of 50%–60%.

Based on the comparison drawn between the CBR values of the geomaterials stabilized with different proportions of pond ash, brick kiln dust and their combination, the optimum dosage/mix proportions have been reported in Table 7 for use in the subgrade of flexible pavements. The optimum mixtures were designated the notations reported in Table 8 for further reference.

### 4.3. Resilient Modulus of Waste Stabilized Geomaterials

The M_R_ of the optimum CLPA, CLBKD and CLPABKD mixtures under unsoaked and soaked conditions, obtained for at 15 stress stages are reported in Table 8. The table shows that the M_R_ is significantly influenced by the stabilization, confining stress, deviator stress and soaking condition. The effect of these factors on M_R_ is discussed in detail in the following sections. The design M_R_ of the subgrade is based on the confining pressure and deviator stress acting at the top of a subgrade as the location receives minimum magnitude of confining stress and maximum magnitude of deviator stress in the subgrade. The deviator stress and confining pressure applicable on the top the subgrade was found closest to predefined stress sequence no. 11, i.e., a confining pressure of 13.8 kPa and a cyclic deviator stress of 12.4 kPa and therefore, was selected for evaluating the change in M_R_ on stabilization with the waste materials [52].

#### 4.3.1. Effect of the Stabilization

**Figure 12:**
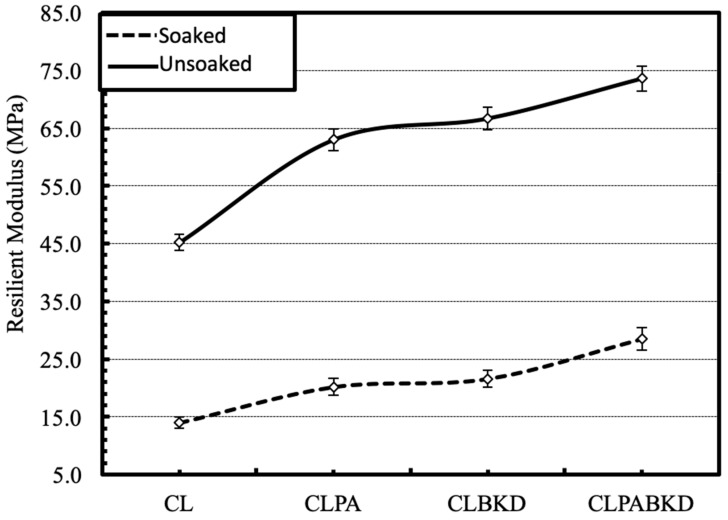
Variation in M_R_ on stabilization of the geomaterial with the optimum contents of pond ash and brick kiln dust under soaked and unsoaked conditions (confining pressure = 13.8 kPa and cyclic deviator stress = 12.4 kPa).

Figure 12 shows the effect of the stabilization of the waste materials on the M_R_ of the geomaterial. The M_R_ of the stabilized geomaterials was higher than the raw geomaterial. The highest M_R_ was achieved for CLPABKD mixture, containing the optimum combination of the waste materials. The M_R_ of CLBKD mixture was moderately higher than CLPA mixture. The M_R_ for CLPA, CLBKD, CLPABKD mixtures increased by 39.1%, 47.2%, and 62.8% respectively, under unsoaked conditions. Similarly, the M_R_ for CLPA, CLBKD, and CLPABKD mixtures increased by 44.3%, 54.4%, and 103.6% respectively, under soaked conditions.

#### 4.3.2. Effect of Deviator Stress

**Figure 13:**
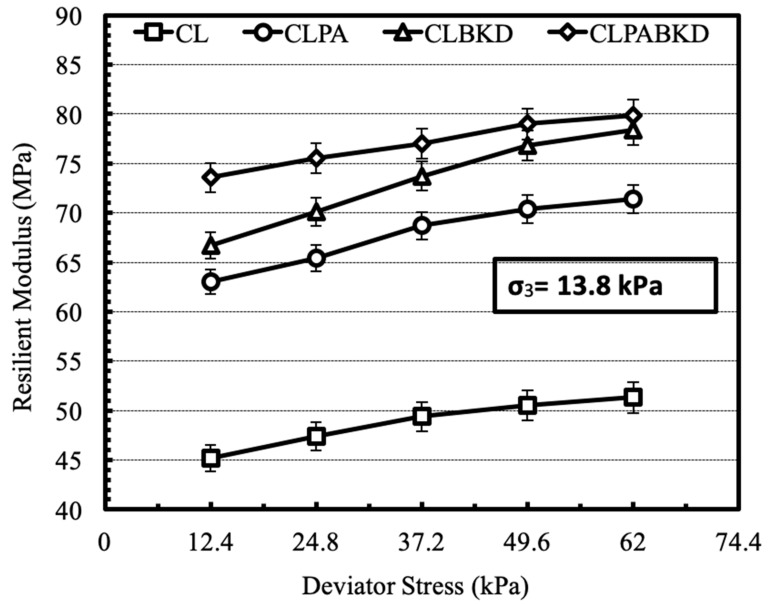
Variation in M_R_ of optimum mixtures (unsoaked) with cyclic deviator stress at a confining pressure of 13.8 kPa.

The effect of cyclic deviator stress on the M_R_ of optimum mixtures (unsoaked), tested at a confining pressure of 13.8 kPa is shown in Figure 13. The M_R_ rises with a rise in the magnitude of cyclic deviator stress. Comparable trends are reported for the M_R_ tests conducted at other confining pressures. The trend may be attributed to strain hardening phenomena wherein increase of deviator stress forces the particles to come together, producing a more compact arrangement of interlocked particles and, therefore, the resilient deformation decreases. A review of previously published work show that strain hardening phenomena is prevalent for geomaterials tested under unsoaked conditions [29].

**Figure 14:**
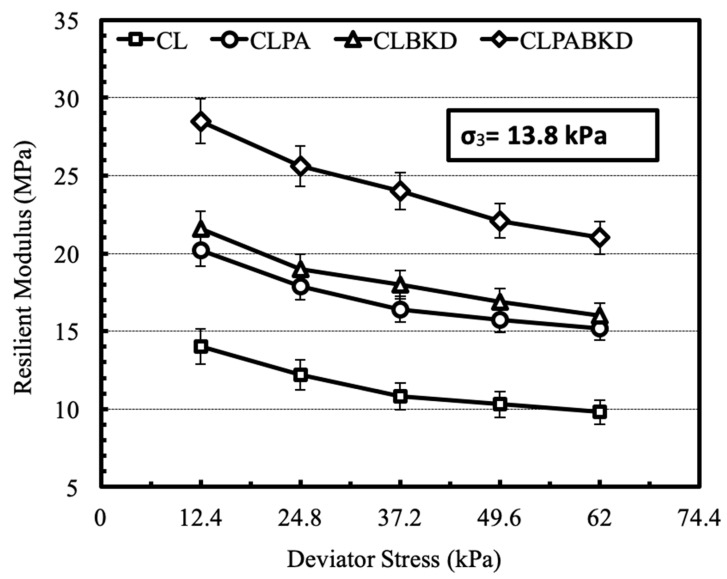
Variation in M_R_ of optimum mixtures (soaked) with cyclic deviator stress at a confining pressure of 13.8 kPa.

The effect of cyclic deviator stress on the M_R_ of optimum mixtures (soaked), tested at a confining pressure of 13.8 kPa is shown in Figure 14. The M_R_ decreases with a rise in the magnitude of cyclic deviator stress. Comparable trends are reported for the M_R_ tests conducted at other confining pressures. The trend may be attributed to strain softening phenomena wherein increase of deviator stress damages the particles and, therefore, the resilient deformation increases. A review of previously published work states that strain softening phenomena is prevalent for geomaterials tested under soaked conditions [26].

#### 4.3.3. Effect of Confining Pressure

**Figure 15:**
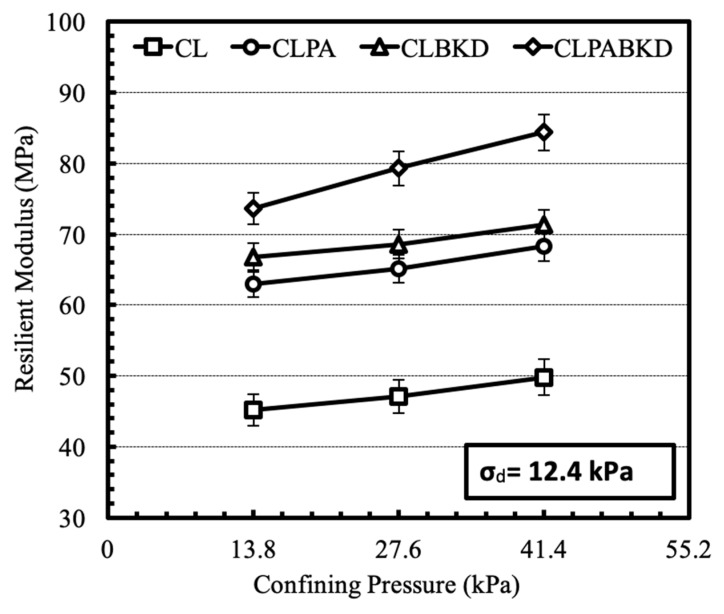
Variation in M_R_ of optimum mixtures (unsoaked) with confining pressure at a cyclic deviator stress of 12.4 kPa.

**Figure 16:**
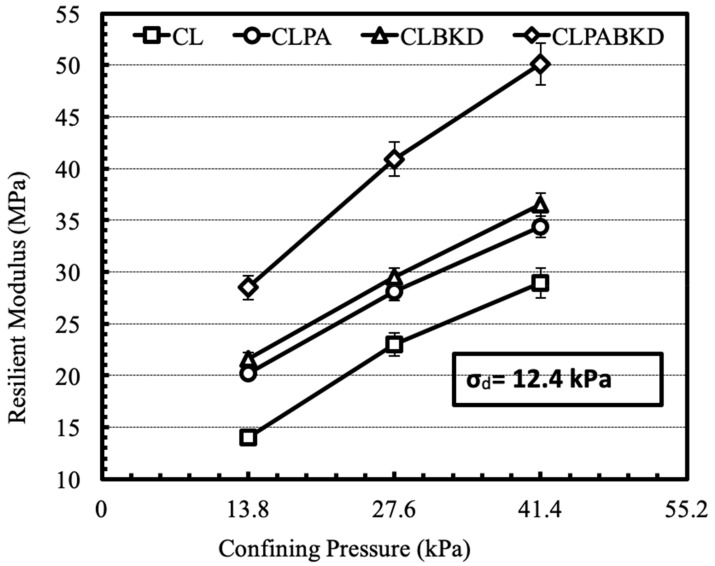
Variation in M_R_ of optimum mixtures (soaked) with confining pressure at a cyclic deviator stress of 12.4 kPa.

The effect of confining pressure on the M_R_ of optimum mixtures, tested at a cyclic deviator stress of 12.4 kPa for unsoaked and soaked samples, is shown in Figure 15 and Figure 16 respectively. The M_R_ increases with a rise in the magnitude of confining stress. Comparable trends are reported for the M_R_ tests conducted at other deviator stresses. This is because, rise in confining pressure lowers lateral strain in the test specimen under repeated loading and therefore, impart higher stiffness to the test specimen. Likewise, trend has been reported in literature for fly ash and cement kiln dust stabilized cohesive geomaterials [26,27].

#### 4.3.4. Effect of Saturation

**Figure 17:**
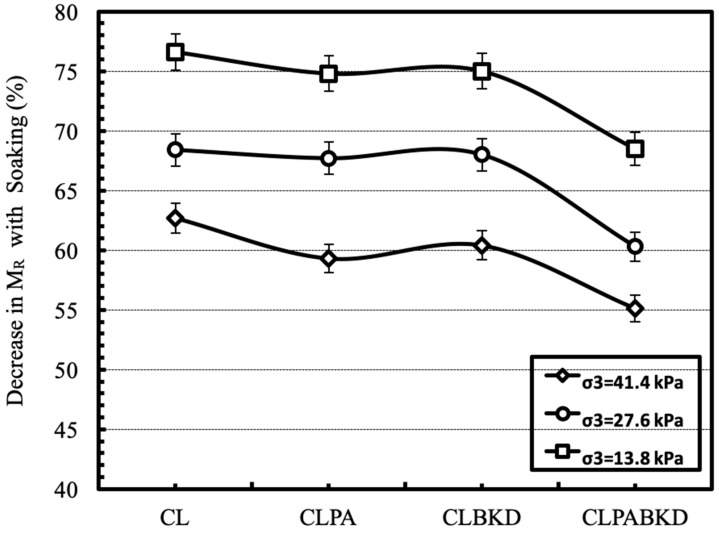
Average decrease in M_R_ (%) with soaking for different optimum mixtures.

For developing a comparison of M_R_ of samples tested in unsoaked and soaked conditions, the decrease in M_R_ was calculated by averaging the cyclic deviator stress for each of the three confining pressures. The average decrease in the percentage of M_R_ due to soaking, tested at different confining pressures have been reported in Figure 17. The soaked M_R_ of the geomaterial and the optimum CLPA, CLBKD and CLPABKD mixtures reported an average drop of 69.2%, 67.3%, 67.8%, and 61.3%, respectively.

#### 4.3.5. Mathematical Models for Prediction of Resilient Modulus

It is well acclaimed that the value of M_R_ is dependent on the magnitude of the stress acting on the specimen under testing. Depending upon the magnitude of applied axle loading and the M_R_ of pavement layer the subgrade is subjected to variable stresses that may not be corresponding to the predefined stress magnitudes listed in AASHTO T 307 [40]. Therefore, based on the M_R_ values obtained under 15 different stress stages the M_R_ response can be modelled using well known equation for prediction of M_R_ as per the field stress conditions. In the present study, the performance of three stress-dependent models recommended by various researchers was compared:

Model 1: a two-parameter model proposed by Witczak and Uzan [53]

(1) $$M_{R}=k_{1}\times {\sigma_{d}}^{k_{2}}$$

Model 2: a two-parameter model proposed by Hicks and Monismith [54]

(2) $$M_{R}=k_{3}\times \theta^{k_{4}}$$

Model 3: a three-parameter model proposed by NCHRP [55] and Puppala et al. [56]

(3) $$\frac{M_{R}}{P_{a}}=k_{5}\times {\left(\frac{\sigma_{3}}{P_{a}}\right)}^{k_{6}}\times {\left(\frac{\sigma_{d}}{P_{a}}\right)}^{k_{7}}$$

where k_1_–k_7_ are the model constants determined through regression analysis. P_a_ corresponds to the atmospheric pressure=100 kPa, σ_1_ corresponds to major principal stress, σ_d_ corresponds to cyclic deviator stress, σ_3_ corresponds to confining pressure, θ corresponds to the sum of three principal stresses known as bulk stress.

The measured resilient modulus values at 15 stress stages were used to calculate the value of model constants k_1_–k_7_ using regression analysis. M_R_ values were back-calculated for the aforementioned 15 stress stages and compared with the measured M_R_ values. Using the results obtained the coefficient of determination (R^2^) for all the models were determined and reported in Table 9 and Table 10 for unsoaked and soaked samples respectively. It was observed that, for all the mixtures, the closest value of R^2^ to 1 was obtained using model 3. The reason for the observation could be due to determination of separate model constants for deviator stress and confining pressure.

The positive value of k_7_ denote strain hardening of the unsoaked optimized mixtures and negative value of k_7_ denote strain softening of the soaked optimized mixtures under cyclic loading. Positive value of k_6_ denotes increase in M_R_ with increase in confining pressure. The behaviour of pond ash and brick kiln dust stabilized geomaterials is in agreement with those of fly ash and cement kiln dust stabilized geomaterials reported in the literature [26,27].

**Figure 18:**
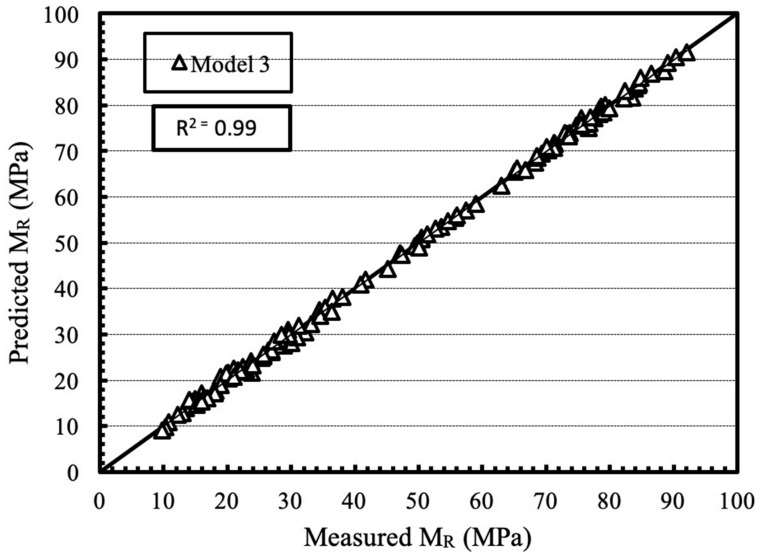
Plot between predicted and measured M_R_ values using model 3 for all the mixtures tested under unsoaked and soaked conditions.

Figure 18 shows the plot between measured and predicted M_R_ values using model 3 for all the mixtures tested under unsoaked and soaked conditions. It was observed that all the results lie close to the equality line.

## 5. Conclusions

The objective of the current study was to evaluate the performance of pond ash and brick kiln dust in stabilization of clay geomaterial for the subgrade of flexible pavements under cyclic loading, based on technical and environmental considerations. The following conclusions can be drawn:

1. Pond ash and brick kiln dust comprise of sand and silt size particles with low specific gravity that do not possess cementitious or pozzolanic properties. Spherical shape of pond ash particles and angular shape of brick kiln dust particles were observed through SEM analysis.
2. With the increase in pond ash or brick kiln dust content, the maximum dry density and plasticity indices of the geomaterial decreases, and the optimum moisture content of the geomaterial increases.
3. Increase in pond ash or brick kiln dust proportion continuously increases the CBR of the geomaterial up to the optimum contents of 30% pond ash, 30% brick kiln dust and a combination of 20% pond ash and 20% brick kiln dust, beyond which the CBR values decreases.
4. Advanced cyclic triaxial testing results show that the M_R_ for the optimized CLPA mixture increase by 39.1% and 44.3%, CLBKD mixture increase by 47.2% and 54.4%, CLPABKD mixture increase by 62.8% and 103.6% for samples tested for unsoaked and soaked conditions.
5. Strain hardening was witnessed in samples tested under unsoaked conditions whereas strain softening was witnessed in samples tested under soaked conditions, during cyclic loading. The M_R_ was reported to increase with increase in confining pressure for all the mixtures. Samples tested under soaked conditions showed a decrease in the range of 60% to 70% as compared to M_R_ of the unsoaked sample. The three-parameter model (Model 3) provided the best fit among different models.
6. The concentrations of toxic elements in pond ash and brick kiln dust were well below the allowable limits for hazardous wastes and thereby are permitted for use as construction materials. A low value of LOI (<5%) assures no risk of spontaneous heating or combustion of the wastes.

## Figures and Tables

**Table 1:** Physical Characteristics of the raw materials.

Characteristics	Pond Ash	Brick Kiln Dust	Clay Geomaterial
Particle size distribution (%)			
Gravel size (20.0–4.75 mm)	0.0	0.0	0.0
Sand size (4.75–0.075 mm)	54.1	72.3	8.9
Silt size (0.075–0.002 mm)	45.9	27.7	72.4
Clay size (<0.002 mm)	0.0	0.0	18.7
Colour	Grey	Red Brown	Brown
Specific Gravity	2.0	2.20	2.76
*D*_60_, mm	0.10	0.18	-
*D*_30_, mm	0.030	0.079	-
*D*_10_, mm	0.015	0.038	-
Uniformity coefficient (C*_U_*)	6.7	4.7	-
Coefficient of curvature (C*_C_*)	0.6	0.9	-
Liquid Limit (%)	-	-	37.3
Plastic Limit (%)	-	-	24.9
Plasticity Index (%)	Non-plastic	Non-plastic	12.4
USCS Classification	SM	SM	CL

**Table 2:** Mix proportions for the geomaterial stabilized with combinations of waste.

Designation of the Mixtures	Pond Ash (%)	Brick Kiln Dust (%)	Clay Geomaterial (%)
PA10BKD10	10	10	80
PA10BKD20	10	20	70
PA10BKD30	10	30	60
PA10BKD40	10	40	50
PA20BKD10	20	10	70
PA20BKD20	20	20	60
PA20BKD30	20	30	50
PA30BKD10	30	10	60
PA30BKD20	30	20	50
PA40BKD10	40	10	50

**Table 3:** Loading sequences used in cyclic triaxial tests.

Sequence No.	Confining Stress (kPa)	Cyclic Deviator Stress (kPa)	Contact Stress (kPa)	Total Cycles
0	41.4	24.8	2.8	500–1000
1	41.4	12.4	1.4	100
2	41.4	24.8	2.8	100
3	41.4	37.3	4.1	100
4	41.4	49.7	5.5	100
5	41.4	62.0	6.9	100
6	27.6	12.4	1.4	100
7	27.6	24.8	2.8	100
8	27.6	37.3	4.1	100
9	27.6	49.7	5.5	100
10	27.6	62.0	6.9	100
11	13.8	12.4	1.4	100
12	13.8	24.8	2.8	100
13	13.8	37.3	4.1	100
14	13.8	49.7	5.5	100
15	13.8	62.0	6.9	100

**Table 4:** Chemical composition of pond ash and brick kiln dust.

Elements as Oxides	Pond Ash (%)	Brick Kiln Dust (%)
SiO_2_	59.2	50.6
Al_2_O_3_	30.9	19.4
Fe_2_O_3_	4.2	11.4
SiO_2_ + Al_2_O_3_ + Fe_2_O_3_	94.3	81.5
CaO	0.94	5.93
K_2_O	1.34	2.23
TiO_2_	1.93	0.93
P_2_O_5_	0.51	2.94
MgO	0.48	1.72
Na_2_O	0.17	0.87
SO_3_	0.12	3.66
MnO	0.04	0.06
LOI	2.1	1.1

**Table 5:** Mechanical characteristics of pond ash and brick kiln dust.

Property	Pond Ash	Brick Kiln Dust
MDD (kN/m^3^)	13.1	16.8
OMC (%)	21.2	18.5
CBR (unsoaked) (%)	9.4	11.2
CBR (soaked) (%)	6.1	7.8
Permeability (cm/s)	5.01 × 10^−5^	7.29 × 10^−5^

**Table 6:** Concentration of toxic elements present in pond ash and brick kiln dust.

Toxic Elements	Concentration (mg/L)		
	Pond Ash	Brick Kiln Dust	USEPA Limits for Hazardous Waste
Chromium	BDL	BDL	5.0
Lead	0.28	0.11	5.0
Arsenic	BDL	0.13	5.0
Mercury	BDL	BDL	0.2
Cadmium	BDL	BDL	1.0
Zinc	0.83	0.85	Not reported
Copper	BDL	BDL	Not reported
Nickel	BDL	BDL	Not reported

BDL: Below detection limit, Detection limit: 0.1 mg/L.

**Table 7:** Notation of the mix proportions for the optimized mixtures.

Optimum Mix Designation	Pond Ash (%)	Brick Kiln Dust (%)	Clay Geomaterial (%)
CLPA	30	0	70
CLBKD	0	30	70
CLPABKD	20	20	60

**Table 8:** Resilient modulus of optimum waste-geomaterial mixtures.

Sequence No.	Confining Stress (kPa)	Cyclic Deviator Stress (kPa)	Resilient Modulus (MPa)							
			Unsoaked				Soaked			
			CL	CLPA	CLBKD	CLPABKD	CL	CLPA	CLBKD	CLPABKD
1	41.4	12.4	49.8	68.3	71.3	84.4	28.9	34.4	36.5	50.1
2	41.4	24.8	53.6	71.3	76.1	88.6	23.8	32.2	33.2	41.7
3	41.4	37.3	55.8	73.3	78.3	89.1	19.0	30.1	31.0	38.0
4	41.4	49.7	57.4	74.7	83.5	90.4	16.0	26.4	27.8	35.3
5	41.4	62.0	59.0	77.6	83.9	92.0	15.0	25.7	27.0	34.6
6	27.6	12.4	47.1	65.1	68.6	79.3	23.0	28.1	29.5	40.9
7	27.6	24.8	50.5	69.3	72.9	82.4	18.2	25.4	26.9	36.4
8	27.6	37.3	52.7	71.2	75.5	84.6	14.6	22.4	23.7	31.2
9	27.6	49.7	54.6	73.2	78.6	84.8	13.5	20.1	21.0	29.7
10	27.6	62.0	56.0	76.7	82.2	86.5	13.0	19.0	19.9	27.4
11	13.8	12.4	45.2	63.0	66.7	73.6	14.0	20.2	21.6	28.5
12	13.8	24.8	47.4	65.4	70.1	75.5	12.2	17.9	19.0	25.6
13	13.8	37.3	49.4	68.7	73.7	77.0	10.8	16.4	18.0	24.0
14	13.8	49.7	50.5	70.4	76.8	79.0	10.3	15.7	16.9	22.1
15	13.8	62.0	51.3	71.4	78.4	79.9	9.8	15.2	16.0	21.0

**Table 9:** Model constants for the optimum mixtures (Unsoaked).

Mixtures	$M_{R}=k_{1}\times {\sigma_{d}}^{k_{2}}$			$M_{R}=k_{3}\times \theta^{k_{4}}$			$\frac{M_{R}}{P_{a}}=k_{5}\times {\left(\frac{\sigma_{3}}{P_{a}}\right)}^{k_{6}}\times {\left(\frac{\sigma_{d}}{P_{a}}\right)}^{k_{7}}$			
	k_1_	k_2_	R^2^	k_3_	k_4_	R^2^	k_5_	k_6_	k_7_	R^2^
CL	36.97	0.10	0.547	20.56	0.20	0.829	0.11	0.67	0.10	0.987
CLPA	52.75	0.08	0.683	36.06	0.14	0.738	0.07	0.85	0.08	0.972
CLBKD	52.44	0.11	0.786	36.57	0.15	0.627	0.06	0.93	0.11	0.967
CLPABKD	69.43	0.05	0.196	34.37	0.19	0.970	0.13	1.05	0.05	0.990

**Table 10:** Model constants for the optimum mixtures (Soaked).

Mixtures	$M_{R}=k_{1}\times {\sigma_{d}}^{k_{2}}$			$M_{R}=k_{3}\times \theta^{k_{4}}$			$\frac{M_{R}}{P_{a}}=k_{5}\times {\left(\frac{\sigma_{3}}{P_{a}}\right)}^{k_{6}}\times {\left(\frac{\sigma_{d}}{P_{a}}\right)}^{k_{7}}$			
	k_1_	k_2_	R^2^	k_3_	k_4_	R^2^	k_5_	k_6_	k_7_	R^2^
CL	50.37	−0.34	0.397	3.01	0.34	0.151	0.51	0.21	−0.34	0.967
CLPA	46.15	−0.21	0.209	3.25	0.41	0.308	0.50	0.35	−0.21	0.975
CLBKD	49.18	−0.21	0.229	3.92	0.38	0.285	0.49	0.36	−0.23	0.974
CLPABKD	68.91	−0.23	0.279	6.14	0.35	0.245	0.45	0.46	−0.23	0.991
